# The reverse TRBV30 gene of mammals: a defect or superiority in evolution?

**DOI:** 10.1186/s12864-024-10632-4

**Published:** 2024-07-19

**Authors:** Fengli Wu, Yingjie Wu, Yuanning Yao, Yuanyuan Xu, Qi Peng, Long Ma, Jun Li, Xinsheng Yao

**Affiliations:** 1 Department of Immunology, Center of Immunomolecular Engineering, Innovation & Practice Base for Graduate Students Education, Zunyi Medical University, Zunyi, China; 2 Queen Mary School, Nanchang University, Nanchang, China

**Keywords:** TRB locus, TRBV30, TCR, CDR3 repertoire, Recombination Signal sequences

## Abstract

At the 3’ end of the C2 gene in the mammalian TRB locus, a distinct reverse TRBV30 gene (named TRBV31 in mice) has been conserved throughout evolution. In the fully annotated TRB locus of 14 mammals (including six orders), we observed noteworthy variations in the localization and quality of the reverse V30 genes and Recombination Signal Sequences (RSSs) in the gene trees of 13 mammals. Conversely, the forward V29 genes and RSSs were generally consistent with the species tree of their corresponding species. This finding suggested that the evolution of the reverse V30 gene was not synchronous and likely played a crucial role in regulating adaptive immune responses. To further investigate this possibility, we utilized single-cell TCR sequencing (scTCR-seq) and high-throughput sequencing (HTS) to analyze TCRβ CDR3 repertoires from both central and peripheral tissues of *Primates* (*Homo sapiens* and *Macaca mulatta*), *Rodentia* (*Mus musculus*: BALB/c, C57BL/6, and Kunming mice), *Artiodactyla* (*Bos taurus* and *Bubalus bubalis*), and *Chiroptera* (*Rhinolophus affinis* and *Hipposideros armige*). Our investigation revealed several novel observations: (1) The reverse V30 gene exhibits classical rearrangement patterns adhering to the ‘12/23 rule’ and the ‘D-J rearrangement preceding the V-(D-J) rearrangement’. This results in the formation of rearranged V30-D2J2, V30-D1J1, and V30-D1J2. However, we also identified ‘special rearrangement patterns’ wherein V30-D rearrangement preceding D-J rearrangement, giving rise to rearranged V30-D2-J1 and forward Vx-D2-J. (2) Compared to the ‘deletional rearrangement’ (looping out) of forward V1-V29 genes, the reverse V30 gene exhibits preferential utilization with ‘inversional rearrangement’. This may be attributed to the shorter distance between the V30 gene and D gene and the ‘inversional rearrangement’ modes. In summary, in the mammalian TRB locus, the reverse V30 gene has been uniquely preserved throughout evolution and preferentially utilized in V(D)J recombination, potentially serving a significant role in adaptive immunity. These results will pave the way for novel and specialized research into the mechanisms, efficiency, and function of V(D)J recombination in mammals.

**Supplementary Information:**

The online version contains supplementary material available at 10.1186/s12864-024-10632-4.

## Introduction

The diversity, specificity, and memory response mechanisms in T and B cells stem from an enormous CDR3 repertoire formed after random recombination of germline V(D)J genes and self-tolerance selection. The theoretical diversity of mouse and human αβ TCR CDR3 repertoires is 10^15^ and 10^18^, respectively [1]. However, in reality, the diversity of the mouse and human peripheral TCR CDR3 repertoires is 2 × 10^8^ and 2.5 × 10^9^, respectively [2, 3]. The source of the evolution of VDJC genes in jawed vertebrates, which emerged massively in a short time, is currently unknown. With the rapid development of sequencing technologies and the increasing annotation of VDJC genes in different species, whether the evolution of adaptive immune response genes is synchronized with that of the species has become a completely new direction for analyzing the differences in immune responses between different species (especially in different mammals) [4–6]. HTS technology can be used to analyze the composition of CDR3 sequences on a large scale from mRNA expression levels, with each sample yielding more than a million unique CDR3 sequences [7, 8]. VDJ (TCR or BCR) sequencing of single cells can be used to characterize the expression levels of two paired-strand mRNAs in individual lymphocytes on a large scale, with more than 5000 T or B cells sequenced per sample [9, 10]. These advancements have led to disruptive breakthroughs in the analysis of the developmental trajectories of T and B cell differentiation, characterization of the CDR3 repertoire, and mechanism of V(D)J rearrangement.

The *H. sapiens* TRB locus consists of 29 forward V gene family, a D1 gene, J1 gene families (containing 6 genes), a C1 gene, a D2 gene, J2 gene families (containing 7 genes), a C2 gene, and a reverse V30 gene [11]. Currently, a total of 14 mammals (in six orders) have fully annotated VDJC sequences in the TRB locus (Supplemental Table S1). Among them, we first annotated the TRB locus of *Chiroptera* and *B. bubalis* [12–14]. Notably, all mammals have evolutionarily acquired a distinct reverse V30 gene at the 3’ end of the C2 gene. However, the origin of the reverse V30 gene, the ways it participates in recombination, the frequency of its usage during rearrangement, and the characteristics of rearranged products have not been elucidated. Moreover, a notable gap exists in the comparative analysis of the evolution and characterization of TRBV genes and corresponding RSS sequences in the limited number of mammalian TRB loci that have been annotated thus far.

The V(D)J recombination of the TRB locus follows the ‘12/23 rule’ and the ‘D-J rearrangement preceding the V-(D-J) rearrangement’ [15, 16]. Additional specific rules also apply, such as the ‘beyond 12/23 rule’ [17] and the ‘V-J direct rearrangement rule’ [18]. Currently, mammalian V(D)J recombination is almost exclusively investigated in humans and mice. Although there is a high degree of concordance between humans and mice, the location of the VDJ genes, differences in RSS quality (https://www.imgt.org/), specific patterns of V(D)J recombination, and advantages of participation in use are not consistent in both human and mouse TR and IG loci. For example, we found that the IGHJ4 gene is frequently expressed in humans but not in mice, indicating that V(D)J recombination may differ across mammals and be correlated with specific adaptive immune responses of T and B cells [19]. In the fully annotated TRB locus of 13 mammals, forward V1-V29 genes undergo rearrangement through the classic ‘deletional rearrangement’, while the reverse V30 gene is recombined through ‘inversional rearrangement’. Taking advantage of scTCR-seq and HTS, our study examined the TCRβ CDR3 repertoires in *H. sapiens* (human) and *M. mulatta* (rhesus monkey), *M. musculus* (BALB/c, C57BL/6, and Kunming mice), *B. taurus* (bovine) and *B. bubalis* (buffalo), and *Chiroptera* (*R. affinis* and *H. armige*), including central tissues (thymus) and peripheral tissues (spleen, blood, and lymph node). The objective of this study was to investigate the evolutionary characteristics of the reverse V30 genes and RSSs in 14 mammals and to focus on the rearrangement modes, frequency of usage, and potential implications of the V30 gene.

## Results

### VDJC genes in the mammalian TRB locus

The locations, names, and numbers of VDJC genes in 14 mammalian TRB locus varied slightly (Supplemental Table S1), among which *H. sapiens*, *M. musculus*, *M. mulatta*, *Macaca fascicularis*, *Canis lupus familiari*, *Felis catus*,* Heterocephalus glaber*, *Oryctolagus cuniculus*, *Rhinolophus ferrumequinum*, and *Mustela putorius furo* had names and arrangements of VDJC gene families consistent with those of V(1–29), D1-J1(1–6)-C1-D2-J2(1–7)-C2, and V30, although there were variations in the number of J gene families in different species. *M. putorius furo* contains a pseudogene known as TRBV30. Additionally, the forward V genes name for *M. musculus* were named V1-V30, and the reverse V gene name was named V31. Various gene families, such as V(1–29), D1-J1(1–6)-C1-D3-J3(1–6)-C3-D2-J2(1–7)-C2, and V30, present in *Ovis aries*, *B. taurus*, *Sus scrofa*, and *B. bubalis*, followed a consistent pattern.

### Evolution of TRBV and RSS sequences, the recombination signal information content (RIC) score of V genes, and the distance from TRBV30 to TRBC2

**Fig. 1:**
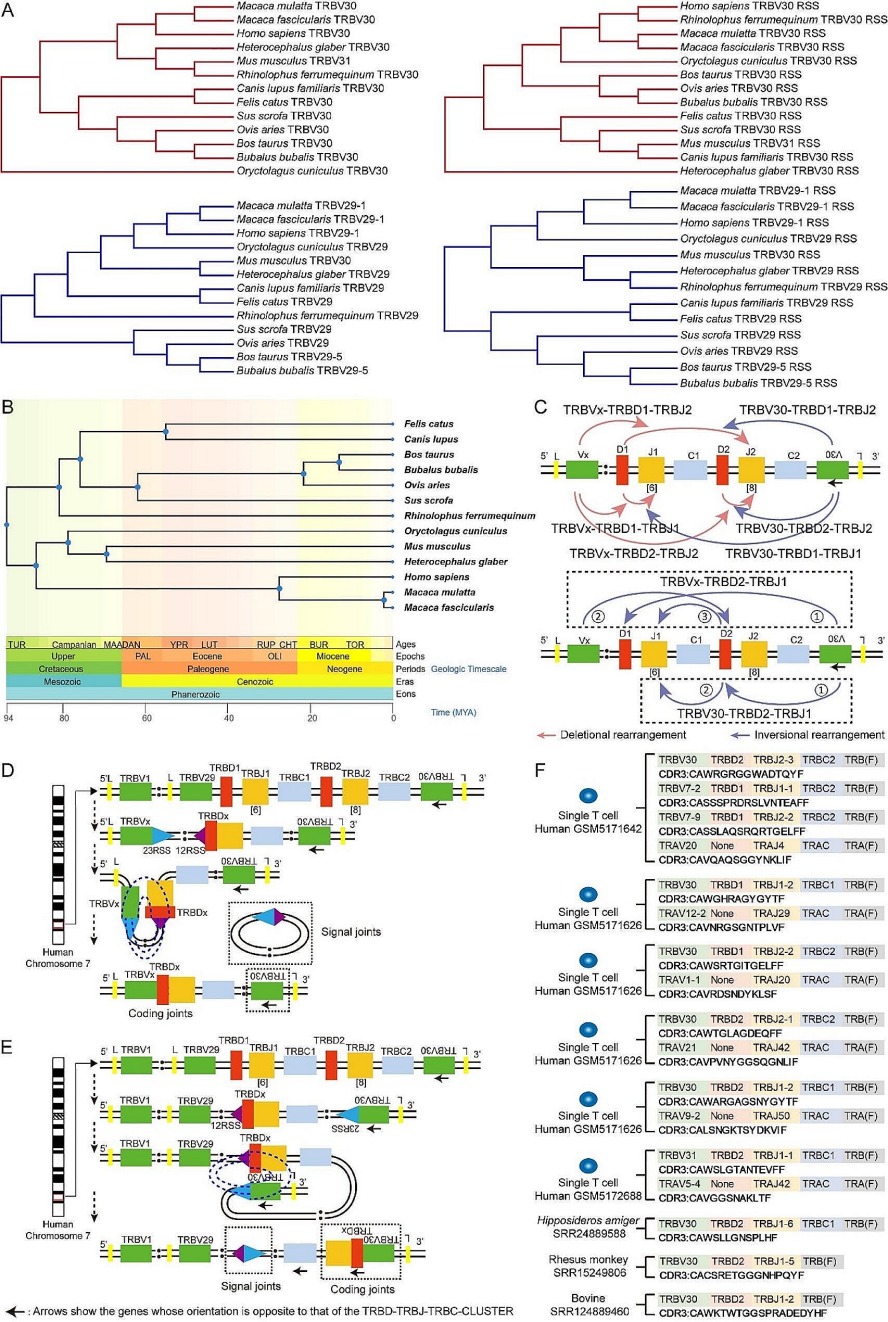
The evolution, modes, and mechanisms involved in rearrangement and examples of VDJ rearranged sequences of V30 genes and V30-RSSs. **(A)** Gene trees of forward V29 and reverse V30 sequences and forward V29-RSS and reverse V30-RSS sequences from 13 mammals. The gene trees were constructed using the Maximum likelihood (ML) method in MEGA7, with bootstrap values deduced from 1000 replicates. **(B)** Species tree of 13 mammals, including *Carnivora*, *Artiodactyla*, *Chiroptera*, *Lagomorph*, *Rodentia*, and *Primates*. **(C)** All possible VDJ recombination pathways involving the human TRB locus are illustrated. **(D)** Illustrative diagram of the deletional rearrangement of forward V genes. **(E)** Illustrative diagram of the inversional rearrangement of reverse V genes. **(F)** A graph displaying sample sequences of multiple rearrangement patterns that were obtained using scTCR-seq and HTS. **Note**: (1) The TRBV30 gene of Domestic ferret is a pseudogene without RIC score, and thus, comparative analysis of the TRBV gene trees was not performed. (2) The mammalian TRB locus contains clusters of V, D, J, and C genes, which are physically arranged in the following order: forward, TRBV gene; TRD1; TRJ1; TRBD2; TRBJ2; TRBC2; reverse, TRBV30 gene (Supplemental Table S1). We used four colors to distinguish between the V, D, J, and C genes in the schematic diagram of the rearrangement mechanism analysis **(**Fig. 1. **C-E)**, and different clusters of genes of the same species were shown in physical order

A comparison of the V30 genes, V29 genes, and RSS sequences of 14 mammals revealed significant variations (Supplemental Table S2-S5, Fig. 1. A, B). Additionally, an examination of the gene trees and mammalian tree revealed that the evolution of V29 genes and V29-RSSs was essentially the same as that of the species. However, notable differences were observed in the evolution of V30 genes and V30-RSSs within the different species.

The reverse V30-RSS RIC score was noticeably lower in 14 mammals, all of which had RIC scores below − 37, with the lowest being in *O. aries* (RIC score < -45). The two exceptions were *M. musculus* and *H. glaber*, which had a slightly higher RIC score for reverse V30-RSS than their mean forward V-RSS RIC score (Supplemental Table S4). For the six mammals in our study, the total mean RIC score for the forward TRBV-RSS was significantly higher than the mean RIC score for the reverse V30-RSS (Supplemental Table S4).

The distance between the TRBV30 and the TRBC2 genes was very close in the same order of species, with the greatest distance occurring in *Artiodactyla* (Supplemental Table S4). Furthermore, the distance between the RSS and the 2nd-CYS (104 C) of the TRBV30 gene was identical across 13 mammals (Supplemental Table S4).

### Analysis of the involvement of reverse TRBV30 in recombination via TCR beta CDR3 sequences

CDR3 repertoires were sequenced using HTS and scTCR-seq in nine mammals. Analysis of CDR3 sequences from each sample revealed that the forward V genes were involved in recombination by deletion, leading to the formation of V-D1J1, V-D1J2, and V-D2J1 sequences. In addition, reverse V30 was involved in recombination by inversion, resulting in the production of the V-D1J1, V-D1J2, and V-D2J1 sequences. However, the particular recombination sequence V30-D2-J1 was detected in each sample. This sequence was analyzed according to the ‘12/23 rule’, suggesting that it originated from the recombination of V30 with D and then with J. This special recombination can also lead to the generation of the unique recombination sequence of forward Vx-D2-J1 **(**Fig. 1. **C-F)**.

### Recombination frequency of reverse TRBV30

**Fig. 2:**
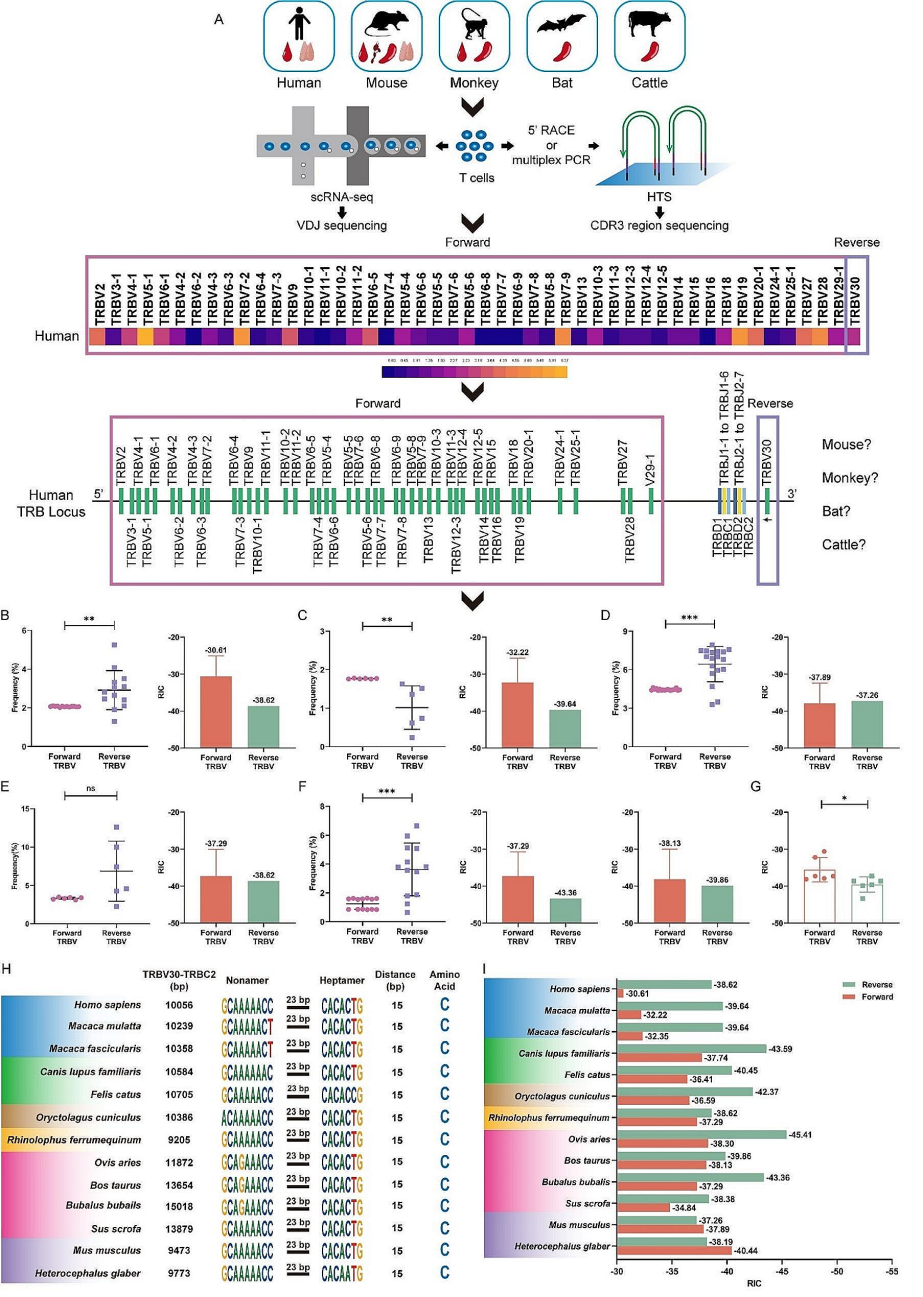
Comparative analysis of gene usage and RIC scores between the forward V1-V29 and the reverse V30 in the *Primates*,* Rodentia*,* Artiodactyla*, and *Chiroptera* central and peripheral TCRβ CDR3 repertoires and comparative analyses of V30-RSS locations and qualities in the 13 mammals. **(A)** Experimental flowchart: collection of central and peripheral samples from humans, rhesus monkeys, mice, bats and cattle; construction of the TCRβ CDR3 repertoire; scTCR-seq and HTS; comparative analysis of forward and reverse V usage frequencies. **(B-G)** Comparative analysis of the frequency of reverse V30 gene use and the average frequency of forward V1-V29 genes use in 9 mammals and corresponding RIC score comparisons. **(B)** Human samples (*n* = 13). **(C)** Rhesus monkey samples (*n* = 6). **(D)** Mouse samples (*n* = 18). **(E)** Bat samples (*n* = 6). **(F)** Cattle samples (*n* = 13). **(G)** Differences between the average RIC scores of the V1-V29 genes and the RIC scores of the reverse V30 gene in 6 mammals. **(H)** Reverse V30-RSS sequences, V30-C2 distances and RSS-C (the 2nd-CYS 104 of the V-REGION) distances in 13 mammals. **(I)** Comparisons between the average V1-V29-23RSS RIC scores and theV30-23RSS RIC score in 13 mammals. **Note**: (1) The name and usage frequency of each TRBV gene family (heatmap) were analyzed for both forward and reverse in humans as an example, and the analysis process was consistent for the other four mammals. (2) The forward TRBV gene usage frequency of each animal was calculated as the average usage frequency according to the number of gene families involved in recombination and compared with the reverse TRBV30 gene usage frequency; moreover, the average RIC scores of the forward TRBV of each mammal were analyzed in comparison with the RIC scores of the reverse TRBV genes. (3) We performed statistical analysis using the Mann-Whitney U test. All statistically significant differences are indicated as *P value < 0.05, **P value < 0.01, and ***P value < 0.001

The number of unique TCR beta CDR3 sequences within each species sample concurred with the statistical comparative analysis of usage frequency for the TRBV gene. Specifically, the usage frequency of reverse V30 genes in humans, mice, cattle, and bats was greater than the average usage frequency of forward V1-V29 genes. Conversely, the six monkey samples displayed a lower usage frequency of 30 genes compared to the average usage frequency of forward V1-V29 genes **(**Fig. 2. **A-F)**.

## Discussion

V(D)J recombination of TCR and BCR and characterization of CDR3 repertoires are fundamental to the study of adaptive immune responses. With the increased annotation of germline VDJC sequences in mammals, we now have the opportunity to examine gene evolution and dominant involvement in rearrangement from a new perspective. These findings provide valuable insights for understanding differences in the adaptive immune responses of T and B cells among different mammals [4–6]. There is a lack of studies reporting whether the recombination of VDJ genes in a forward or reverse manner affects their usage frequency. Although the results of our large-scale CDR3 sequence analysis revealed that the reverse V gene had an advantage in recombination, further research on the combined effects of factors such as distance and RSS quality is necessary. In our completed VDJC annotation of mammalian TR and IG locus, such as bats and buffaloes, we found that the TRB loci also contained a unique reverse TRBV30 gene [12, 13, 20]. In the fully annotated TRB locus of 14 mammals, we observed that the forward V29 and V29-RSSs gene trees were consistent with the corresponding species tree **(**Fig. 1. **A**,** B)**. However, we identified significant locational variations in the V30 gene and V30-RSS gene trees in *R. ferrumequinum*, *Canis lupus familiaris*, *O. cuniculus*, and *S. scrofa*. Specifically, the V30 gene in *R. ferrumequinum* appeared to be most similar to that in *M. musculus*, while the V30 gene in *S. scrofa* was most similar to that in several other species of *Artiodactyla*. Interestingly, the V30-RSS in *R. ferrumequinum* was identical to that in humans. Additionally, the V30-RSSs in *S. scrofa* were more closely related to those in *Carnivora*. Furthermore, the reverse V30 gene in *M. putorius furo* evolved into a pseudogene, and its V30-RSSs exhibited extremely low quality and were unable to be used to calculate the RIC score. Compared to the TRBV gene tree of the corresponding species tree, the V30 genes and V30-RSSs gene trees demonstrated asynchronization. In addition, the basic composition and location of the reverse TRBV30 genes in each mammal were determined by chromosome annotation, and these genes were fixed in each species. Our research focused on the composition of V genes and RSSs on chromosomes in 13 mammalian species and possible differences between different species, but excluded the analysis of possible differences in gene mutations at the mRNA level. The results showed that the V genes and the corresponding RSSs in mammals were conserved, and both the forward and reverse V genes and the corresponding RSSs showed convergence, but there were some differences between them. The quality of V genes and corresponding RSSs on chromosomes is closely related to the usage frequency of their participation in rearrangement [21, 22]. Recently, Zhang et al. demonstrated that not only RSS quality but also the direction of V genes involved in recombination, such as forward ‘deletional rearrangement’ (looping out) and ‘inversional rearrangement’, significantly affected the usage frequency of V genes [23]. This discovery will provide new insight into the evolutionary and response variability of T cells among different mammals. It is important to emphasize that the relationship between immune genes (VDJC) and species evolution, which we have carried out for the first time, is only a preliminary analysis of the possible relationship between the evolution of TRBV and RSS sequences and species evolution from the 13 mammalian species (including six orders) that have been completed thus far. Based on the large number of mammals, more mammalian VDJC genes annotations are needed in the future to comparatively analyze the relationship between the evolution of immune gene and species evolution to explore the differences in adaptive immune responses in different mammals. Moreover, the effects of differences in reverse TRBV30 gene evolution among different mammals on the mechanism of V(D)J recombination and TCR CDR3 repertoire bias during thymic T cell development need to be further investigated.

We further analyzed the qualities of all forward and reverse V gene RSSs using RIC scores. Obviously, the reverse V30-23RSS RIC score was lower than the average forward V1-V29-23RSS RIC score, except for *M. musculus and H. glaber*, which exhibited similar scores. This indicated a lower level of conservation and a greater frequency of mutations in the reverse V30-RSSs. At the same time, the distance between V30 and C2 remained relatively consistent across different species within the same order, indicating a degree of coherence between its evolution and the evolution of the corresponding species. Specifically, *Artiodactyla* (including sheep, bovines, pigs, and buffaloes) exhibited the most significant distance (Fig. 1. B, Supplemental Table S4). These results imply substantial convergence in the ancestral VDJ genes of different mammalian orders, making them potential candidate genes for species evolution divergence points.

In the mammalian TRB locus, the forward 3’ V1-V29 RSSs (7-23-9) and the 5’D RSSs (9-12-7) underwent rearrangement through ‘deletion’. The two coding ends were ligated to form a coding joint, which remained on the chromosome. The two signal ends were ligated to form a signal joint and then looped out of the chromosome (Fig. 1. C, D). The reverse 5’V30 RSSs (9-23-7) and the 5’D RSSs (9-12-7) participated in the rearrangement via ‘inversion’, remaining on the chromosome after the joining of RSSs **(**Fig. 1. **C**,** E)**. The regulatory factors, such as RAG, Ku, DNA-dependent protein kinase (DNA-PK), XRCC4, and terminal deoxynucleotidyl transferase (TdT), are consistent in both types of rearrangement [16]. The rearrangement modes and efficiency of TRB forward and reverse V genes are currently unknown. We studied the clonotype sequences of the TCRβ CDR3 repertoires in 56 samples from the central and peripheral tissues of 9 mammals (including four orders) and investigated the rearrangement modes of V30. In each sample, there were classical rearrangement modes of V30 that follow conventional rules such as the ‘12/23 rule’ [15] and the ‘D-J preceding the V-DJ rule’ [16], as well as the ‘novel rearrangement mode’ where V30-D rearrangement precedes D-J **(**Fig. 1. **C**,** F)**. The classical mode contributed to the production of rearranged V30-D2J2, V30-D1J1, and V30-D1J2, while it caused the rearrangement of V30-D2-J1 and forward Vx-D2-J1 **(**Fig. 1. **C**,** F)**.

The ‘novel rearrangement mode’ is similar to the ‘beyond 12/23 rule’ [17] and the ‘V-J direct rearrangement rule’ in TRB [18]. It remains unclear whether this mode is a ‘blemish’ in VDJ precise rearrangements or serves as a ‘supplement’ to the classical rearrangement mode, compensating for the impossible direct D2 and J1 rearrangement in the TRB locus under the traditional mode and expanding the diversity of the TCRβ CDR3 repertoire. However, further research investigating more physiological and pathological models of mammals is needed. Additionally, in our previous scTCR-seq of T-cell samples, we also found simultaneous production of reverse V30-D2J2 and forward Vx-D1J1 rearrangement sequences, leading to ‘allelic inclusion’ rearrangements on the same chromosome. Further research is required to study the frequency and significance of these rearrangements [24].

The preferential utilization of VDJ subfamily genes is influenced by various factors, including location [10], RSS quality [25], and the distance between the RSS location and V or J [26]. However, there is a lack of research on the impact of the rearrangement mode on utilization. In the TCRβ CDR3 repertoires of 56 samples, we found that the ‘inversional rearrangement’ of the reverse V30 gene in *H. sapiens*, *M. musculus*, *Chiroptera* and *Artiodactyla* was preferentially utilized compared to the ‘deletional rearrangement’ of the forward V1-V29 genes **(**Fig. 2. **A-G)**. This phenomenon could not be explained by the RSS quality, as the V30 RIC score was lower than the V1-V29 RIC scores. The mechanism behind preferential utilization may be closely related to the shorter V30-D distance compared to that of V1-V29-D and the ‘inversional rearrangement’ instead of the ‘deletional rearrangement’ of the V1-V29 genes. However, in the six peripheral samples from *M. mulatta*, the V30 gene usage frequency was lower than the average usage frequency of the forward V1-V29 genes. This discrepancy may be attributed not only to the significant difference in low V30-23RSS (RIC score = -39.64) and high average V1-V29-23RSS (RIC score = -32.22) but also to other factors in *M. mulatta* that could prevent its use in rearrangement **(**Fig. 2. **H**,** I)**.

## Conclusion

The TRB locus in mammals has evolved unique reverse V30 genes and RSSs. Its gene trees showed inconsistency with the forward V genes and RSSs, as well as the corresponding species. This finding suggested that the reverse V30 was active in evolution and might play an essential regulatory role in adaptive immune evolution. The ‘novel rearrangement pattern’ of V30 could compensate for the inability of mammals to recombine V30-D2-J1 and forward Vx-D2-J1, thereby increasing the diversity of the CDR3 repertoire. The ‘inversion’ mode and ‘short-distance’ in the rearrangement of reverse V30 may be related to its preferential utilization. In summary, 13 annotated mammalian TRB loci currently have a consistent reverse V30 gene. Our large-scale analysis of the CDR3 repertoires obtained from HTS and scTCR-seq in humans, mice, monkeys, bats, buffalo, and cattle suggested that the reverse V30 gene was not retained at the 3’ end of the C region of the chromosome due to an evolutionary defect but rather that an evolutionary advantage may have caused it to be retained through a higher frequency of recombination. This greatly increased the diversity of the TCR V30-CDR3 repertoires and played an important role in specific epitope response effects. These findings will contribute to further research on the specific mechanisms, efficiency, and functions of VDJ recombination in mammals.

## Materials and methods

### Evolutionary characteristics and RIC scores of the TRBV gene in mammals

The nucleotide sequences of the V30 and V29 genes and the RSS sequences of 14 mammals were obtained from the IMGT database [14]. Bat and buffalo sequences were annotated by our research group [12, 13].

Clustal Omega, provided by EMBL-EBI (http://www.ebi.ac.uk), was used to perform a comparative analysis of reverse V30 genes (named V31 in mice) and RSS sequences, as well as forward V29 genes (named V30 in mice) and RSS sequences in 13 mammals. The TRBV30 gene of domestic ferret (*M. putorius furo*) is a pseudogene without RIC score, and thus, comparative analysis was not performed. TRBV gene trees and RSS sequence trees were constructed using the Maximum likelihood (ML) method in MEGA7, with bootstrap values deduced from 1000 replicates [27, 28].

The species tree of the 13 mammals was constructed using the TimeTree of Life resource (TToL5) [29] (https://www.timetree.org), which can retrieve published studies and divergence times between species, the timeline of species evolution beginning with the origin of life, and the timetree for a given evolutionary group at the desired taxonomic rank.

WebLogo 3 (htttps://weblogo.threeplusone.com/creat.cgi) was used to generate base composition plots of the Heptamer and Nonamer sequences present in the reverse TRBV30 RSS sequences of the 13 mammals. This tool calculated the distance between the TRBV30 gene and the TRBC2 gene and the distance between the RSS and the TRBV30 gene. WebLogo 3 generates sequence logos, graphical representations of the patterns within a multiple sequence alignment. Each logo consists of stacks of letters, and the overall height of each stack indicates the sequence conservation at that position (measured in bits), whereas the height of the symbols within the stack reflects the relative frequency of the corresponding amino or nucleic acid at that position [30].

The RSS site (htttps://www.itb.cnr.it) was utilized to analyze and calculate the V-RSS RIC scores of 13 mammals, and comparative analysis and statistical tests were conducted. The RSS site can predict the RSS quality of any given sequence and output the RIC score [21]. This algorithm calculates the theoretical recombination potential of an RSS using a statistical model that assigns a score based on the contribution of each nucleotide within the heptamer, 12 or 23 spacer, or nonamer [22].

### scTCR-seq and HTS were utilized to analyze the modes of rearrangement and frequencies of usage of the V gene in the TCRβ CDR3 repertoires of 7 mammals

#### Animals

All experiment procedures were carried out in accordance with the Ethics Committee of Zunyi Medical University (the bats and mice project was approved under permit number (2018)2-261, and the buffaloes and cattle project was approved under permit number ZMU21-2203-111). All experiment procedures were conducted under the guidelines of the ARRIVE guidelines. Both BALB/c and Kunming mice and bats were euthanized using the cervical dislocation method. The buffaloes and cattle were sourced from an abattoir that followed all the ethical standards of animal slaughter in Nanning City, Guangxi Province, China. The buffaloes and cattle were approved for research purposes, having successfully passed local health and veterinary inspections. Our animal experiments did not involve humans, rhesus monkeys, and C57BL/6 mice. The HTS and ScRNA-seq data of humans, rhesus monkeys, and C57BL/6 mice were downloaded from the ENA and the NCBI databases.

#### HTS sequencing of mammalian TCRβ CDR3 repertoires

Four human thymus samples (ERZ1694549, ERZ1694551, ERZ1694560, and ERZ1694569) and four human peripheral blood samples (ERZ1694578, ERZ1694579, ERZ1694580, and ERZ1694581) were collected from infants aged 7, 52, 107, and 156 days, respectively. DNA was extracted from thymus tissues and peripheral blood mononuclear cells (PBMCs), and the TCRβ CDR3 repertoires were constructed using multiplex PCR. Sequencing was conducted on the Illumina platform, and the relevant sample information was obtained from the article (DOI: 10.1016/j.dib.2021.106751) [31]. The analysis data can be found in the EMBL-EBI public database under the accession number PRJEB41936 (https://www.ebi.ac.uk/ena/browser/view/PRJEB41936) (Supplemental Table S6).

We collected spleen and thymus samples from 3 female BALB/c mice aged 2 months and thymus samples from 3 Kunming mice. Total RNA was extracted from each sample, and the 5’ RACE method was used to construct the TCRβ CDR3 repertoires. Sequencing was performed using an Illumina NovaSeq 6000 platform. The raw data of each sample were uploaded to the NCBI database (accession numbers: PRJNA906203 and PRJNA982279) (https://www.ncbi.nlm.nih.gov/bioproject/?term=PRJNA906203, https://www.ncbi.nlm.nih.gov/bioproject/?term=PRJNA982279), including 3 spleen samples from BALB/c mice (SRR22437999, SRR22437998, and SRR22437997), 3 thymus samples from BALB/c mice (SRR22438002, SRR22438001, and SRR22438000), and 3 thymus samples from Kunming mice (SRR24908413, SRR24908412, and SRR24908411) (Supplemental Table S7).

Blood sample was obtained from a 5-year-old healthy female Chinese rhesus monkey. PBMCs were isolated using density gradient centrifugation, and total RNA was extracted. The TCRβ CDR3 repertoire was constructed using 5’ RACE, and sequencing was conducted using the Illumina HiSeq 2000 platform. The basic information of the sample can be found in a previously published article (DOI:10.1371/journal.pone.0182733) [32]. The analysis data were obtained from the NCBI database (accession number: PRJNA38923) (https://www.ncbi.nlm.nih.gov/bioproject/PRJNA389234) (Supplemental Table S8).

We collected 3 *R. affinis* and 3 *H. armiger* specimens from Guizhou, China. Spleen tissue samples were obtained from each bat, and total RNA was extracted. The 5’ RACE method was employed to construct the TCRβ CDR3 repertoires, and sequencing was performed using the Illumina HiSeq 1500 platform. The raw data for each sample were uploaded to the NCBI database (accession numbers: PRJNA877449 and PRJNA982392) (https://www.ncbi.nlm.nih.gov/bioproject/?term=PRJNA877449, https://www.ncbi.nlm.nih.gov/bioproject/?term=PRJNA982392), including 3 *R. affinis* spleen samples (SRR21464509, SRR21464508, and SRR21464510) (Supplemental Table S9) and 3 *H. armiger* spleen samples (SRR24889588, SRR24889587, and SRR24889586) (Supplemental Table S10).

We collected spleen samples from 6 buffaloes and 7 bovines, and DNA was extracted from each sample. Multiplex PCR was used to construct the TCRβ CDR3 repertoires, and sequencing was performed using the MGISEQ-2000RS platform. The raw data of each sample were uploaded to the NCBI database (accession numbers: PRJNA908273, PRJNA982388, and PRJNA982389) (https://www.ncbi.nlm.nih.gov/bioproject/?term=PRJNA908273, https://www.ncbi.nlm.nih.gov/bioproject/?term=PRJNA982388, https://www.ncbi.nlm.nih.gov/bioproject/?term=PRJNA982389), including 6 *B. bubalis* spleen samples (SRR22523497, SRR24889447, SRR24889446, SRR24889445, SRR24889444, and SRR24889443) (Supplemental Table S11) and 7 *B. taurus* spleen samples (SRR24889460, SRR24889459, SRR24889458, SRR24889457, SRR24889456, SRR24889455, and SRR24889454) (Supplemental Table S12).

#### ScTCR-seq of mammalian TCRβ CDR3 repertoires

Five human peripheral blood samples (GSM171626, GSM171627, GSM5171634, GSM171635, and GSM171642) were obtained from five healthy donors aged 39, 71, 55, 68, and 41 years, respectively. PBMCs were isolated using density gradient centrifugation, and single-cell sequencing was performed on the Illumina HiSeq 3000 platform. The basic information of the samples is available in the published article (DOI:10.1038/s41467-022-29175-x) [33]. The analysis data were obtained from the NCBI database (accession number: GSE168859) (https://www.ncbi.nlm.nih.gov/geo/query/acc.cgi? acc = GSE168859) (Supplemental Table S6).

Lymph node samples (GSM5172690, GSM5172691, and GSM5172698), three spleen samples (GSM5172688, GSM5172689, and GSM5172696) and three blood samples (GSM5172686, GSM5172687, and GSM5172694) were collected from 3 C57BL/6 tumor-bearing mice aged 6–8 weeks. Immunomagnetic bead negative selection was used to pre-enrich total T cells. Single-cell sequencing was performed on the Illumina HiSeq 4000 platform. The basic sample information is derived from a published article (DOI:10.1084/jem.20201329) [34]. The analysis data were obtained from the NCBI database (accession number: GSE168944) (https://www.ncbi.nlm.nih.gov/geo/query/acc.cgi? acc = GSE168944) (Supplemental Table S7).

One spleen sample (SRR15249798) and two PBMC samples (SRR15249806 and SRR15249810) from a rhesus monkey were used. FACS was employed to stimulate nonproliferating T cells (SRR15249814) and proliferating T cells (SRR15249812). The 5’RACE method was used for the construction of the TCRβ CDR3 repertoire. Single-cell sequencing was performed on the Illumina NextSeq 500 platform. The basic information of the samples was obtained from a published article (DOI: 10.4049/jimmunol.2100824) [35]. The analysis data were sourced from the NCBI database (accession number: PRJNA746267) (https://www.ncbi.nlm.nih.gov/bioproject/?term=PRJNA746267) (Supplemental Table S8).

#### Rearrangement modes and usage frequency of V genes in the TCRβ CDR3 repertoire

HTS sequencing of mammalian TCRβ CDR3 repertoires: The raw data were analyzed using MiXCR (version 3.0.13). The TRB VDJC gene background reference library in MiXCR was obtained from the VDJC gene sequences of humans, mice, rhesus monkeys, and bovines in the IMGT database [14], as well as the VDJC gene sequences of *R. affinis* and *B. bubalis* annotated by our research group [12, 13]. MiXCR is a robust bioinformatic tool that is capable of processing B or T cell immune repertoire data from raw sequences into quantified clonotypes and encompasses three key steps: alignment, annotation, and clonotyping [36]. The unique feature of MiXCR is the use of a specially designed JSON structured format to store a comprehensive reference library of V/D/J/C gene sequences and markup. In paired-end sequencing analysis, MiXCR aligns both read and aggregate information from both alignments to achieve high V and J gene assignment accuracy.

The data output from MiXCR were analyzed as follows: (1) Deletion of unproductive sequences, identified by the presence of “_” and “*” in the CDR3 amino acid sequence. (2) Deletion of sequences that do not start with “C” and end with “F” in the CDR3 amino acid sequence, as these are unproductive sequences. (3) The removal of sequences in the TCRβ CDR3 repertoire where the V and J genes are pseudogenes or ORFs. (4) Counting and analysis of the usage frequency of each V gene subfamily in the Productive Clonotype sequences of each sample.

ScTCR-seq of mammalian TCRβ CDR3 repertoires: Cell Ranger (human: version 2.1.1; mouse: version 2.1.0; rhesus macaque: version 4.0.0) was used to analyze the raw data of each sample and perform quality control and screening on the output data. (1) Analyze the output “filtered_contig_annotations.CSV” file and the sequence of “FALSE” in “is_cell” was deleted. (2) The sequence of “FALSE” in “high_confidence” was deleted. (3) Other sequences in “chain” that were not “TRB” were deleted. (4) The TRBV sequence of the open reading frame and pseudogenes in “v_gene” were deleted. (5) The sequence of “None” in “d_gene” was deleted. (6) The sequence of “None” in “c_gene” was deleted. (7) The sequence of “FALSE” in “full_length” was deleted. (8) The “None” and “FALSE” “productive” sequences to obtain all productive TRBV gene sequences were deleted. (9) Counting and analysis of the usage frequency of each TRBV gene subfamily in the Productive Clonotype sequences of each sample.

We calculated the usage frequency of each TRBV gene family in the total sequence of the TCRβ CDR3 repertoire after quality control screening of each sample. Statistical comparisons were made to analyze the difference between the average frequency of the forward V1-V29 genes (V1-V30 for *M. musculus*) and the reverse V30 gene (V31 for *M. musculus*) for each mammal. At the same time, we calculated the mean RSS-RIC of the forward V1-V29 genes for each mammal and compared their differences with the RSS-RIC of the reverse V30 gene for each mammal.

### Statistical analyses

The Mann-Whitney U test was used to analyze the differences between each sample and each group of samples. The significance threshold was set at a *P* value = 0.05. All statistically significant differences were indicated as *P value < 0.05, **P value < 0.01, and ***P value < 0.001.

### Electronic supplementary material

Below is the link to the electronic supplementary material.

Supplementary Material 1

## Data Availability

All the raw data in this study have been uploaded to the NCBI database under the accession numbers PRJNA906203 (https://www.ncbi.nlm.nih.gov/bioproject/?term=PRJNA906203), PRJNA982279 (https://www.ncbi.nlm.nih.gov/bioproject/?term=PRJNA982279), PRJNA877449 (https://www.ncbi.nlm.nih.gov/bioproject/?term=PRJNA877449), PRJNA982392 (https://www.ncbi.nlm.nih.gov/bioproject/?term=PRJNA982392), PRJNA908273 (https://www.ncbi.nlm.nih.gov/bioproject/?term=PRJNA908273), PRJNA982388 (https://www.ncbi.nlm.nih.gov/bioproject/?term=PRJNA982388), and PRJNA982389 (https://www.ncbi.nlm.nih.gov/bioproject/?term=PRJNA982389).

## Declarations

### Declaration of ARRIVE guidelines

We declared that this study was reported in accordance with the ARRIVE guidelines (https://arriveguidelines.org). (1) Research design: Our research subjects were *Primates* (*H. sapiens* and *M. mulatta*), *Rodentia* (*M. musculus*), *Artiodactyla* (*B. taurus* and *B. bubalis*), and *Chiroptera* (*R. affinis* and *Hipposideros armiger*). (2) The sample sizes were as follows: 13 *H. sapiens* (human), 6 *M. mulatta* (rhesus monkey), 6 *M. musculus* (BALB/c, C57BL/6, and Kunming mice) samples, 18 *R. affinis* and *H. armige* samples, and 13 *B. taurus* (bovine) and *B. bubalis* (buffalo) samples. Our study did not include a control group, and we compared each group of animals. (3) Randomization: We used a random method to include the study animals. (4) Experimental animals and procedure: Four human thymus samples and four peripheral blood samples were collected from infants aged 7, 52, 107, and 156 days. DNA was extracted from thymus tissues and peripheral blood mononuclear cells (PBMCs), and the TCRβ CDR3 repertoire was constructed using multiplex PCR. Sequencing was conducted on the Illumina platform, and the relevant sample information was obtained from the article (DOI: 10.1016/j.dib.2021.106751). The other five peripheral blood samples were obtained from five healthy donors aged 39, 71, 55, 68, and 41 years. PBMCs were isolated using density gradient centrifugation, and single-cell sequencing was performed on the Illumina HiSeq 3000 platform. The basic information of the samples is available in the published article (DOI:10.1038/s41467-022-29175-x). The mouse samples from our group were collected from 3 female BALB/c mice aged 2 months and 3 Kunming mice. Total RNA was extracted from each sample, and the 5’RACE method was used to construct the TCRβ CDR3 repertoire. Sequencing was performed using an Illumina NovaSeq 6000 platform. In another mouse sample from 3 C57BL/6 tumor-bearing mice aged 6–8 weeks, immunomagnetic bead negative selection was used to pre-enrich total T cells. Single-cell sequencing was performed on the Illumina HiSeq 4000 platform. The basic sample information is derived from a published article (DOI:10.1084/jem.20201329). One spleen sample and two PBMC samples from a rhesus monkey were used. FACS was employed to stimulate nonproliferating T cells (SRR15249814) and proliferating T cells (SRR15249812). The 5’RACE method was used for the construction of the TCRβ CDR3 repertoire. Single-cell sequencing was performed on the Illumina NextSeq 500 platform. The basic information of the samples was obtained from a published article (DOI: 10.4049/jimmunol.2100824). One blood sample was obtained from a 5-year-old healthy female Chinese rhesus monkey. PBMCs were isolated using density gradient centrifugation, and total RNA was then extracted. The TCRβ CDR3 repertoire was constructed using 5’RACE, and sequencing was conducted using the Illumina HiSeq 2000 platform. The basic information of the sample can be found in a previously published article (DOI:10.1371/journal.pone.0182733). The *Chiroptera* samples were collected from 3 *R. affinis* and 3 *H. armiger* in Guizhou, China. Spleen tissue samples were obtained, and total RNA was extracted. The 5’RACE method was employed to construct the TCRβ CDR3 repertoires, and sequencing was performed using the Illumina HiSeq 1500 platform. *Artiodactyla* samples were collected from 6 buffaloes and 7 bovines, and DNA was extracted from spleen samples. Multiplex PCR was used to construct the TCRβ CDR3 repertoires, and sequencing was performed using the MGISEQ-2000RS platform. (5) Results: All the sequencing data were used for the analysis of the TCRβ CDR3 repertoire. (6) Statistical methods: SPSS (version 29.0) was used for statistical analysis, and we performed statistical analysis using the Mann-Whitney U test. All statistically significant differences are indicated as **P* value < 0.05, ***P* value < 0.01, and ****P* value < 0.001.
