# Supplementary material for: The reverse TRBV30 gene of mammals: a defect or superiority in evolution?
Source: BMC Genomics. 2024 Jul 19;25:705. doi: 10.1186/s12864-024-10632-4 (PMC11264764; doi:10.1186/s12864-024-10632-4)
Supplement: Supplementary file 1 — Supplementary Material 1 [file 12864_2024_10632_MOESM1_ESM.docx]

**Supplemental Table S1. VDJC locations and names on 14 mammalian TRB gene locus.**

| **Mammal name** | **TRB locus** | **Forward TRBV** | **TRBD-TRBJ-TRBC** | **Reverse TRBV** | **Reference** |
| --- | --- | --- | --- | --- | --- |
| Human (*Homo sapiens*), *Primates* | chromosome 7 | V(1-29) | D1-J1(1-6)-C1-D2-J2(1-7)-C2 | V30 | IMGT |
| House mouse (*Mus musculus*), *Rodentia* | chromosome 6 | V(1-30) | D1-J1(1-7)-C1-D2-J2(1-7)-C2 | V31 | IMGT |
| Rhesus monkey (*Macaca mulatta*), *Primates* | chromosome 3 | V(1-29) | D1-J1(1-6)-C1-D2-J2(1-7)-C2 | V30 | IMGT |
| Crab-eating macaque (*Macaca fascicularis*), *Primates* | chromosome 3 | V(1-29) | D1-J1(1-6)-C1-D2-J2(1-7)-C2 | V30 | IMGT |
| Dog (*Canis lupus familiaris*), *Carnivora* | chromosome 16 | V(1-29) | D1-J1(1-6)-C1-D2-J2(1-6)-C2 | V30 | IMGT |
| Domestic cat (*Felis catus*), *Carnivora* | chromosome A2 | V(1-29) | D1-J1(1-6)-C1-D2-J2(1-6)-C2 | V30 | IMGT |
| Naked mole-rat (*Heterocephalus glaber*), *Rodentia* |  | V(1-29) | D1-J1(1-6)-C1-D2-J2(1-7)-C2 | V30 | IMGT |
| Rabbit (*Oryctolagus cuniculus*), *Lagomorpha* |  | V(1-29) | D1-J1(1-6)-C1-D2-J2(1-6)-C2 | V30 | IMGT |
| *Rhinolophus ferrumequinum*, *Chiroptera* | chromosome 26 | V(1-29) | D1-J1(1-6)-C1-D2-J2(1-9)-C2 | V30 | Zhou(2, 3) |
| Sheep (*Ovis aries*), *Artiodactyla* | chromosome 4 | V(1-29) | D1-J1(1-6)-C1- D3-J3(1-6)-C3-D2-J2(1-7)-C2 | V30 | IMGT |
| Bovine (*Bos taurus*), *Artiodactyla* | chromosome 4 | V(1-29) | D1-J1(1-6)-C1- D3-J3(1-5)-C3-D2-J2(1-7)-C2 | V30 | IMGT |
| Pig (*Sus scrofa*), *Artiodactyla* | chromosome 18 | V(1-29) | D1-J1(1-7)-C1- D3-J3(1-7)-C3-D2-J2(1-6)-C2 | V30 | IMGT |
| Buffalo (*Bubalus bubalis*), *Artiodactyla* | chromosome 8 | V(1-30) | D1-J1(1-6)-C1- D3-J3(1-6)-C3-D2-J2(1-7)-C2 | V30 |  |
| [Domestic ferret](https://www.imgt.org/IMGTrepertoire/index.php?section=LocusGenes&repertoire=locus&species=ferret&group=TRB#locus_main) (*Mustela putorius furo*), *Carnivora* |  | V(1-30) | D1-J1(1-6)-C1-D2-J2(1-6)-C2 | V30 | IMGT |

**Note:** The TRBV30 gene of Domestic ferret *(Mustela putorius furo)* is a pseudogene without RIC score, and thus it was not performed comparative analysis in the TRBV gene tree.

**Supplemental Table S2. Reverse TRBV30 gene sequences in 14 mammals**

| **Species** | **Accession numbers** | **Gene** | **TRBV Sequence** |
| --- | --- | --- | --- |
| *Homo sapiens* | L36092 | TRBV30 | tctcagactattcatcaatggccagcgaccctggtgcagcctgtgggcagcccgctctctctggagtgcactgtggagggaacatcaaaccccaacctatactggtaccgacaggctgcaggcaggggcctccagctgctcttctactccgttggtattggccagatcagctctgaggtgccccagaatctctcagcctccagaccccaggaccggcagttcatcctgagttctaagaagctccttctcagtgactctggcttctatctctgtgcctggagtgt |
| *Mus musculus* | X03277 | TRBV31 | gctcagactatccatcaatggccagttgccgagatcaaggctgtgggcagcccactgtctctggggtgtaccataaaggggaaatcaagccctaacctctactggtactggcaggccacaggaggcaccctccagcaactcttctactctattactgttggccaggtagagtcggtggtgcaactgaacctctcagcttccaggccgaaggacgaccaattcatcctaagcacggagaagctgcttctcagccactctggcttctacctctgtgcctggagtct |
| *Macaca mulatta* | NW_001114291 | TRBV30 | tctcagactgttcatcaatggccagcgaccctggtgcagcctgcgggcagcccgctttctctggagtgcactgtggagggaacatcaaaccccaacctatactggtaccgacaggctgcaggcaggggcctccagctgctcttctactccattggtgttgaccagatcagctctgaggtgccccagaatctctcagcctccaggccccaggacaggcggttcatcctgagttctaagaagctcctcctcagtgactcaggcttctatctctgtgcctggagtgt |
| *Macaca fascicularis* | IMGT000075 | TRBV30 | tctcagactgttcatcaatggccagcgaccctggtgcagcctgcgggcagcccgctttctctggagtgcactgtggagggaacagcaaaccccaacctatactggtaccgacaggctgcaggcaggggcctccagctgctcttctactccattggtgttgaccagatcagctctgaggtgccccagaacctctcagcctccaggccccaggacaggcagttcatcctgagttctaagaagctcctcctcagtgactcaggcttctatctctgtgcctggagtgt |
| *Canis lupus familiaris* | IMGT000005 | TRBV30 | gctcagactatccaccaaaggccgcttgccagggtgcagcttgtgggcagcctgctctccctggaatgtaccgtgcagggggcatcgagcccttatctctactggtaccggcagtccctgggaggtgcgccccagctactcttctcctcattaagtgttacccagatagtccctgagacaccgcacaacttcacagcctccaggccccagaacggccagttcatcctgagttctaagaagctccttctcagtgactctggcttctacctctgcgcctggagtct |
| *Felis catus* | IMGT000037 | TRBV30 | gctcagaccatccaccaatggccacctgtcagggtgcagcttgtgggcagcccgctctccctggagtgcatcgtgaagggggaatcaaacccttatctatattggtacctgcaggccgagggagggcccccccagctgctcttctactccctaaatattcaccaggtagaccctgaggcaccacggaacttcacagcctccaggccccaggacggccagttcatcttgagttctcagaagctcctcctcagtgactctggcttctacctctgcgcctggggtct |
| *Heterocephalus glaber* | IMGT000070 | TRBV30 | gctcagactatccatcaatggccagttaccaaggtgcagcttgtgggcagccccctctctctggagtgcatcgtgaaggggaaatcaagccccaatctatactggtaccagcaggtagcaggaggggccctccagctgctcttctactccatcggtgttggcgaggtgttctctgaggtactcagaaacctctcagcctccagaccccaggatggccagttcatcctgagctctaagaaactgctcctcaatgactctggcttctacctctgtgcctggagcct |

**Refer to Supplemental Table S2**

| **Species** | **Accession numbers** | **Gene** | **TRBV Sequence** |
| --- | --- | --- | --- |
| *Oryctolagus cuniculus* | IMGT000032 | TRBV30 | gctcagaccattcaccagtggccagctttcagggtgcaacttgtgggcagcccactctccctgcagtgcaccgtgaagggtgtctcaagccccaacctgtactggtaccggcaggctgcagaggggtctctccaggctctcttcttctccattggtgtcggccaggtggaccctgaggggccccagaacctgtcagcctccagaccccaggacgaccagttcatcctgagctctccgaagctcctcctcagtgactcgggcttctacctctgtgcctggagtct |
| *Rhinolophus ferrumequinum* |  | TRBV30 | gctcagaccatccatcagtggccacctacaagggtgcaggctgtggacagtccactctctctgaactgcactgtgaaggggacgtcaagccccaacctatactggtaccggcaggccgcaggaggggccctccagctgcttttctattccattagtattggggacgtagcctctgagggtgaccagaactggaatgcgtccaggccccaggacggccacttcatcctgagcactgagaagctgctcctcagccactctggcttctacctctgtgcctggagtct |
| *Ovis aries* | AM420900 | TRBV30 | gctcagaccatccatcaatggccatccaccagggtgcagcctgcaggcagcccgctctctctggagtgcaccgtgaaggggacatcaagccccaacctgtactggtaccggcaggaggcaggggggagcctccagctgctcttctcctctgttggtgttaaccagatagagcctagggagttccagaacttcgaagcttccaggccccaggacggccagttcaccctgagttctaagaagctgcagctcaataactctggcttctacctctgcgcctggagtct |
| *Bos taurus* | [IMGT000084](https://www.imgt.org/ligmdb/result.action?accessionNumber=IMGT000084) | TRBV30 | actcagaccatccatcagtggccatccacgagggtgcagcctgcaggcagcccgctctctctggagtgcaccgtgaaggggacatcaaaccccaccctgtactggtaccggcaggaggcaggggggagcctccagcagctcttctactctgttagtgctggccagatagaacctagggagttccagaacttcaaagcttccaggccccaggacggccagtttaccctgagttctaagaagctgcagctcaacaactccggcttctacttctgtgcctggagtct |
| *Sus scrofa* | IMGT000039 | TRBV30 | gctcagaccatccatcaatggccagctaccagggtgcagcttgtgggcagcgccctctccctggagtgcactgtgaagggggtatcaagccccagcctatactggtacaggcaggccaccggcgggggggaccttcagctgctcttctactccattggcgttgaccagaaagatcctgaaaagctccagaacttcaacgcctccaggccccaggatggactgttcatcctgagttctacgaagctcctgctcagcaactctggcttctacctctgcgcctggagtct |
| *Bubalus bubalis* |  | TRBV30 | gctcagaccatccatcaatggccatccaccagggtgcagcctgcaggcagcccgctctctctggagtgcaccgtgaaggggacatcaaaccccagcctgtactggtaccggcaggaggcaggggggagcctccagcagctcttctactctgttagtgctggccagatagaacctagggagttccagaacttcaaagcttccaggccccaggacggccagtttaccctgagttctaagaagctgcagctcaataactcgggcttctacctctgcgcctggagtct |
| *Mustela putorius furo* | [IMGT000023](https://www.imgt.org/ligmdb/result.action?accessionNumber=IMGT000023) | TRBV30 | gctccaaccatccaccaagggccgcctgtcagcgtgcagcctataggcagcctgcttgccctggaatgcactgtgaaggggacatcagtcccttatctgtattggtacctgcggtccccgggagggaccccagcgttgctcttctcctctgtaaatgttgaccagatagttcgtgagacaccgcagaacttcacagccctcaggcccacggatggccagttcatcctgaattctgagaagctcctgctcagtgactccggcttctatctccgcgcctggagtgtcg |

**Supplemental Table S3. The forward TRBV29 gene sequences in 14 mammals**

| **Species** | **Accession numbers** | **Gene** | **TRBV Sequence** |
| --- | --- | --- | --- |
| *Homo sapiens* | L36092 | TRBV29-1 | agtgctgtcatctctcaaaagccaagcagggatatctgtcaacgtggaacctccctgacgatccagtgtcaagtcgatagccaagtcaccatgatgttctggtaccgtcagcaacctggacagagcctgacactgatcgcaactgcaaatcagggctctgaggccacatatgagagtggatttgtcattgacaagtttcccatcagccgcccaaacctaacattctcaactctgactgtgagcaacatgagccctgaagacagcagcatatatctctgcagcgttgaaga |
| *Mus musculus* | AE000664 | TRBV30 | agtgtcctcctctaccaaaagccaaacagggacatctgtcaaagtggcacttcactgaaaatccagtgtgtggctgacagtcaagttgtttcgatgttttggtaccaacagttccaggaacagagcttgatgctcatggcaactgcaaatgaaggctctgaagccacatacgagagtggattcaccaaggacaagtttccaatcagccggccaaacctaacattctcaacgttgacagtgaacaatgcaaggcctggagacagcagtatctatttctgtagttctagaga |
| *Macaca mulatta* | NW_001114291 | TRBV29-1 | agtgctgtcatctctcaaaagccaagcagggatgtctgtcaacgtggaacctccgtgaagatccagtgtcaagtcgatagccaagtcaccatgatgttctggtaccgtcagcaacctggacagagcatgacactgattgcaactgcaaatcagggctctgaggccacatatgagagtggatttgtcattgacaagtttcccatcagtcgcccaaacctaacattctcaactctaactgtgagcaacacgagccctgaagacagcagcatatacctctgcagcgttgaaga |
| *Macaca fascicularis* | IMGT000075 | TRBV29-1 | agtgctgtcgtctctcaaaagccaagcagggatgtctgtcaacgtggaacctccgtgacgatccagtgtcaagtcgatagccaagtcaccatgatgttctggtaccgtcagcaacctggacagagcatgacactgattgcaactgcaaatcagggctctgaggccacatatgagagtggatttgtcattgacaagtttcccatcagtcgcccaaacctaacattctcaactctgactgtgagcaacacgagccctgaagacagcagcatatacctctgcagcgttgaaga |
| *Canis lupus familiaris* | IMGT000005 | TRBV29 | ggagctcttgtctctcaaaagccgcgcagggacatctgtcaacgtgggacctccattaccatccactgtgaggtcgatacccaagtcaccttgatgttctggtaccgtcagctcccaggacagagcttgatactgattgcaaccgcaaaccagggtgcagaggccacctacgaaagtggatttaccagggagaagtttcccatcagccgccgaaccctaatgttctccactctgactgtgagcaacctgagcctcgaagacaccagctcttacttctgcagcgctagaga |
| *Felis catus* | IMGT000037 | TRBV29 | agcgctctcctctctcagaagccacacagagacatctgtcaacgtgggacctccgtgacaatccactgtgaggtcgatatccagttcaccttaatgttctggtaccatcagctcccaggacagagcttggtgctgatggcaaccacaaaccagggtctggaggccacttacgaacatggatttaccaaggacaagtttcccatcagccgcccaaccctagtgttctcaactatgaccataagcaacgtgagccttgaagatagcagcttttacttctgtagtgccggaga |
| *Heterocephalus glaber* | IMGT000070 | TRBV29 | ggtgttatccttcatcaaaagtcaaccagagaaatctgtcaaagtgggacctccatgacaatccagtatcaggctgacatccaggtatccctgatcttctggtaccatcaggccccaggacagagcttggtgctgattgcaactggaaatcaaggctctgaggccacatatgagaatagattttccaaggacaaagtttctcatcagccacccaaacctaacattcacaactctggctatgattagtacatgccccaaagatagcagcttctactactgctgtgctggaga |

**Refer to Supplemental Table S3**

| **Species** | **Accession numbers** | **Gene** | **TRBV Sequence** |
| --- | --- | --- | --- |
| *Oryctolagus cuniculus* | IMGT000032 | TRBV29 | ggtgttctcgtctctcaaaagccaatcagggacatctgtcagcgtggaaactccatcatgatccagtgtcaggtcgatgttcaagcgtccctgatgttctggtaccgtcagctcccgggacagagcttgatactgatcgcaactgcaaatcagggttctgaggccacgtatgagagtggattcaccaaggacaagtttcccatcactcgtcctaacctgacattttcaaccctcactgtgagtgatgtgagccctgaagacaacggcttatacctctgcagctttggaga |
| *Rhinolophus ferrumequinum* |  | TRBV29 | ggtactctcgtctctcaaaagccaagcagggacatctgtcaacgcgggacctccgtgacgatcgagtgtcaagtagacagccaaataaacttcatgttctggtaccgtcagctcccaggagggagcttgacactgattgcaactgcgaatcagggctccggggccacctatgaaagtggatttaccaaggacaaatttcccatcagccgcccaaacctaatgttctccaacctgactgtgaccaacgtgcgccccgaagacagcagcttttacttctgcagcgttggaga |
| *Ovis aries* | AM420900 | TRBV29 | ggtgctctcctctctcaaaagccaagcagggccatccgtcaacgtgggacctccatgatgatcgagtgtcaggtcgatagccagctcaccttgatgtactggtaccgtcagcttccaggacagagcttggtgctgatggctactgccaatcagggctccaaggctacttacgagagtgggttcactgaggacaagtttcccattagccgcccgaacctggcgttctcaactctgactgtgagcaacgcgagctccgaagatagcagctcttatttctgcagtgctggaga |
| *Bos taurus* | [IMGT000084](https://www.imgt.org/ligmdb/result.action?accessionNumber=IMGT000084) | TRBV29-5 | ggtgctctcctctctcaagagccaagcagggccatccgtcaacgcgggacctccatgacgatcgagtgtcaagtcgatagccagctcacctggatgtactggtaccgtcagcttccaggacagagcttggtgctgatggctactgccaatcagggctccaaggctacttatgagagtgggtttactgaggacaagtttcccattgaccgcccgaaactggagttctcaaccctgactgtgagcaacgcgagctccgaagacagtagctcttatttctgcagtgctggaga |
| *Sus scrofa* | IMGT000039 | TRBV29 | ggtgttctcctctctcaaaagccaagcagagacatctgccaacgcgggacctctgtgatgatccagtgccaggtcgatagcgagttcacctacatgtactggtaccgtcagcttctaggacaaagcttgacactgatggcagctgtgggtcgggacttcgaggccacttatgagagtggatttaccaaggaaaagtttcccattagccgcccaaacctgatgttctcaattctgaccgtgagcaacgtgagctctgaagacagcagctcctacttctgcagcgctggaga |
| *Bubalus bubalis* |  | TRBV29-5 | ggtgctctcctctctcaaaagccaagcagggccatctctcaacgcgggacctccgtgatgatcgagtgtcgtcaggttgatagccagctcacctggatgtactggtaccgtcagcttccaggacagagcttggtgctgatggctactgccaatcagggctccaaggctacttacgagagggggtttactgaggccaagtttcccattgaccgcccaaaactggagttctcaactctgactgtgagcaacgtgagctccgaagacagcagctcttatttctgcagtgctgcaga |
| *Mustela putorius furo* | [IMGT000023](https://www.imgt.org/ligmdb/result.action?accessionNumber=IMGT000023) | TRBV29 | agcactctcctctctcaagagccacgcagggacatctgtcagtgcgggacctccatgacgatccagtgtgagactgatacccaagtctccttaatgtactggtaccgtcagctcccaggacagagcctgatactgattgcaactgcaaaccagggcatggaggccacttatgaaagtggatttaccaaagagaaatttcccatcagccgcccaaccttaacgttctccagtctgaccgtgaacaacatgagcttcgaagatagcagcttttacctctgcagtgctgaaga |

**Supplemental Table S4.** **RSSs, RIC scores and V30-C2 distances of reverse TRBV30 gene in 14 mammals**

| **Species** | **Accession numbers** | **Gene** | **23RSS sequence** | **RIC scores** | **V30-C2**  **Distance(bp)** |
| --- | --- | --- | --- | --- | --- |
| *Homo sapiens* | L36092 | TRBV30 | cacactgagctgggtggggcagacatctgtgcaaaaacc | -38.62 | 10056 |
| *Mus musculus* | X03277 | TRBV31 | cacactgagtagggtggggcagacatctgtgcaaaaacc | -37.26 | 9473 |
| *Macaca mulatta* | NW_001114291 | TRBV30 | cacactgagctgggtggggcagacatctgtgcaaaaact | -39.64 | 10239 |
| *Macaca fascicularis* | IMGT000075 | TRBV30 | cacactgagctgggtggggcagacatctgtgcaaaaact | -39.64 | 10358 |
| *Canis lupus familiaris* | IMGT000005 | TRBV30 | cacactgagtgggtggggggagacatctgtgcaaaaaac | -43.59 | 10584 |
| *Felis catus* | IMGT000037 | TRBV30 | cacaccgagccgggtgaggtagacatctgtgcaaaaacc | -40.45 | 10705 |
| *Heterocephalus glaber* | IMGT000070 | TRBV30 | cacaatgtaccgggcagagcagacatctgtgcaaaaacc | -38.19 | 9773 |
| *Oryctolagus cuniculus* | IMGT000032 | TRBV30 | cacactgagctgggtggggcagagaactgtacaaaaacc | -42.37 | 10386 |
| *Rhinolophus ferrumequinum* |  | TRBV30 | cacactgagctgggtggggcagacatctgtgcaaaaacc | -38.62 | 9205 |
| *Ovis aries* | AM420900 | TRBV30 | cacactgcactgggtggggcagacatccgtgcagaaacc | -45.41 | 11872 |
| *Bos taurus* | [IMGT000084](https://www.imgt.org/ligmdb/result.action?accessionNumber=IMGT000084) | TRBV30 | cacactgcgctgggtggggcagacatctgtgcagaaacc | -39.86 | 13654 |
| *Sus scrofa* | IMGT000039 | TRBV30 | cacactgcgtccggtggggcagacatctgtgcaaaaacc | -38.38 | 13879 |
| *Bubalus bubalis* |  | TRBV30 | cacactgcactgggtggggcagacatctgtgcagaaacc | -43.36 | 15018 |
| *Mustela putorius furo* | [IMGT000023](https://www.imgt.org/ligmdb/result.action?accessionNumber=IMGT000023) | TRBV30 | cacagaggtgggtggggcagacatctgtacaaaaccc | FAIL | 10778 |

**Supplemental Table S5. RSSs, RIC scores of forward TRBV29, and the average RIC scores of forward TRBV (1-29) in 14 mammals**

| **Species** | **Accession numbers** | **Gene** | **23RSS** | **RIC scores of TRBV29** | **RIC scores of forward TRBV (1-29)** |
| --- | --- | --- | --- | --- | --- |
| *Homo sapiens* | L36092 | TRBV29-1 | cacagtgcggggcacagatcaaagatctgagcaagaacc | -34.82 | -30.61 |
| *Mus musculus* | AE000664 | TRBV30 | cacagtgctggttgcaagggagaaatctcagcgagaact | -50.42 | -37.89 |
| *Macaca mulatta* | NW_001114291 | TRBV29-1 | cacagtgctgggcacagatcaaagatctgagcaagaacc | -33.46 | -32.22 |
| *Macaca fascicularis* | IMGT000075 | TRBV29-1 | cacagtgctgggcacagatcaaagatctgagcaagaacc | -33.46 | -37.29 |
| *Canis lupus familiaris* | IMGT000005 | TRBV29 | cacagcgcccagcacggatcaaagatctgaacaagaacc | -38.30 | -37.29 |
| *Felis catus* | IMGT000037 | TRBV29 | cacagtactcagcacggatcagaggtctgagcaagaacc | -40.97 | -38.13 |
| *Heterocephalus glaber* | IMGT000070 | TRBV29 | cacagtgctgggcacagatcaaagaactaagcaagaact | -43.69 | -32.35 |
| *Oryctolagus cuniculus* | IMGT000032 | TRBV29 | cacagtgctgggcacagatcagagatctgagcaagaacc | -32.14 | -37.74 |
| *Rhinolophus ferrumequinum* |  | TRBV29 | cacagtgctgggcacagatcaaagatctgagcaagaact | -35.47 | -36.41 |
| *Ovis aries* | IMGT000042 | TRBV29 | cacagtgctgggcacggatcaagggtctcagcaagaacc | -38.87 | -40.44 |
| *Bos taurus* | [IMGT000084](https://www.imgt.org/ligmdb/result.action?accessionNumber=IMGT000084) | TRBV29-5 | cacagtgctgggcacagttcaagggtctcagcaagaacc | -39.67 | -36.59 |
| *Sus scrofa* | IMGT000039 | TRBV29 | cacagtgctgtgcacagatcaaaggtctcaacaagaacg | -43.24 | -38.3 |
| *Bubalus bubalis* |  | TRBV29-5 | cacagtgctgggcacagatcaagggtctcagcaagaacc | -37.81 | -34.84 |
| *Mustela putorius furo* | [IMGT000023](https://www.imgt.org/ligmdb/result.action?accessionNumber=IMGT000023) | TRBV29 | cacagtccccggcacagatcaaagatctgagcaagaacc | -41.86 | -36.13 |

**Supplemental Table S6. Basic information and unique TCRβ CDR3 sequences analyzed for all human samples**

| **Accession Number** | **Species** | **Tissue** | **Condition** | **Starting material** | **Library Preparation approach** | **Sequencing** | **Analysis Sequence Clonetype** |
| --- | --- | --- | --- | --- | --- | --- | --- |
| ERZ1694549 | Human | Thymus | Congenital heart defects | DNA | multiplex PCR | HTS | 96616 |
| ERZ1694551 | Human | Thymus | Congenital heart defects | DNA | multiplex PCR | HTS | 81013 |
| ERZ1694560 | Human | Thymus | Congenital heart defects | DNA | multiplex PCR | HTS | 61308 |
| ERZ1694569 | Human | Thymus | Congenital heart defects | DNA | multiplex PCR | HTS | 55729 |
| ERZ1694578 | Human | Blood | Congenital heart defects | DNA | multiplex PCR | HTS | 33817 |
| ERZ1694579 | Human | Blood | Congenital heart defects | DNA | multiplex PCR | HTS | 31686 |
| ERZ1694580 | Human | Blood | Congenital heart defects | DNA | multiplex PCR | HTS | 46336 |
| ERZ16945481 | Human | Blood | Congenital heart defects | DNA | multiplex PCR | HTS | 35756 |
| GSM5171626 | Human | Blood | Healthy | RNA | Nested PCR | ScTCR-seq | 12394 |
| GSM5171627 | Human | Blood | Healthy | RNA | Nested PCR | ScTCR-seq | 13473 |
| GSM5171634 | Human | Blood | Healthy | RNA | Nested PCR | ScTCR-seq | 8059 |
| GSM5171635 | Human | Blood | Healthy | RNA | Nested PCR | ScTCR-seq | 7223 |
| GSM5171642 | Human | Blood | Healthy | RNA | Nested PCR | ScTCR-seq | 50743 |

**Note:** It is important to note that the raw sequencing data, basic information and the unique TCRβ CDR3 sequences for all research samples have been uploaded to the NCBI database. Multiple laboratories, including our own, sequenced and uploaded the samples to the shared database. Researchers and readers have access to the shared data for each sample and can analyze and cite it. However, please include detailed information, such as the accession number, when citing the data.

**Supplemental Table S7. Basic information and unique TCRβ CDR3 sequences analyzed for all mice samples**

| **Accession Number** | **Species** | **Tissue** | **Condition** | **Starting material** | **Library Preparation approach** | **Sequencing** | **Analysis Sequence Clonetype** |
| --- | --- | --- | --- | --- | --- | --- | --- |
| GSM5172690 | C57BL/6 mice | Lymph node | Cancer | RNA | Nested PCR | scRNA-seq | 7226 |
| GSM5172691 | C57BL/6 mice | Lymph node | Cancer | RNA | Nested PCR | scRNA-seq | 7803 |
| GSM5172698 | C57BL/6 mice | Lymph node | Cancer | RNA | Nested PCR | scRNA-seq | 3088 |
| GSM5172688 | C57BL/6 mice | Spleen | Cancer | RNA | Nested PCR | scRNA-seq | 10158 |
| GSM5172689 | C57BL/6 mice | Spleen | Cancer | RNA | Nested PCR | scRNA-seq | 6669 |
| GSM5172696 | C57BL/6 mice | Spleen | Cancer | RNA | Nested PCR | scRNA-seq | 5367 |
| GSM5172686 | C57BL/6 mice | Blood | Cancer | RNA | Nested PCR | scRNA-seq | 6856 |
| GSM5172687 | C57BL/6 mice | Blood | Cancer | RNA | Nested PCR | scRNA-seq | 5947 |
| GSM5172694 | C57BL/6 mice | Blood | Cancer | RNA | Nested PCR | scRNA-seq | 2777 |
| SRR22438002 | BALB/c mice | Thymus | Healthy | RNA | 5’RACE | HTS | 795330 |
| SRR22438001 | BALB/c mice | Thymus | Healthy | RNA | 5’RACE | HTS | 538438 |
| SRR22438000 | BALB/c mice | Thymus | Healthy | RNA | 5’RACE | HTS | 465501 |
| SRR24908413 | Kunming mice | Thymus | Healthy | RNA | 5’RACE | HTS | 740204 |
| SRR24908412 | Kunming mice | Thymus | Healthy | RNA | 5’RACE | HTS | 846904 |
| SRR24908411 | Kunming mice | Thymus | Healthy | RNA | 5’RACE | HTS | 678187 |
| SRR22437999 | BALB/c mice | Spleen | Healthy | RNA | 5’RACE | HTS | 416140 |
| SRR22437998 | BALB/c mice | Spleen | Healthy | RNA | 5’RACE | HTS | 582692 |
| SRR22437997 | BALB/c mice | Spleen | Healthy | RNA | 5’RACE | HTS | 766508 |

**Supplemental Table S8. Basic information and unique TCRβCDR3 sequences analyzed for all rhesus monkey samples**

| **Accession Number** | **Species** | **Tissue** | **Condition** | **Starting material** | **Library Preparation approach** | **Sequencing** | **Analysis Sequence Clonetype** |
| --- | --- | --- | --- | --- | --- | --- | --- |
| SRR5647486 | Rhesus monkey | Blood | Healthy | RNA | 5’RACE | HTS | 140355 |
| SRR15249798 | Rhesus monkey | Spleen | Healthy | RNA | Nested PCR | scRNA-seq | 5223 |
| SRR15249806 | Rhesus monkey | Blood | Healthy | RNA | Nested PCR | scRNA-seq | 5497 |
| SRR15249810 | Rhesus monkey | Blood | Healthy | RNA | Nested PCR | scRNA-seq | 6487 |
| SRR15249812 | Rhesus monkey | Blood | Healthy | RNA | Nested PCR | scRNA-seq | 21819 |
| SRR15249814 | Rhesus monkey | Blood | Healthy | RNA | Nested PCR | scRNA-seq | 29906 |

**Supplemental Table S9. Basic information and unique TCRβCDR3 sequences analyzed for all *Rhiolophus Affnis* samples**

| **Accession Number** | **Species** | **Tissue** | **Condition** | **Starting material** | **Library Preparation approach** | **Sequencing** | **Analysis Sequence Clonetype** |
| --- | --- | --- | --- | --- | --- | --- | --- |
| SRR21464510 | *Rhiolophus affnis* | Spleen | Healthy | RNA | 5’RACE | HTS | 2097 |
| SRR21464509 | *Rhiolophus affnis* | Spleen | Healthy | RNA | 5’RACE | HTS | 13050 |
| SRR21464508 | *Rhiolophus affnis* | Spleen | Healthy | RNA | 5’RACE | HTS | 26564 |

**Supplemental Table S10. Basic information and unique TCRβCDR3 sequences analyzed for all *Hipposideros armige* samples**

| **Accession Number** | **Species** | **Tissue** | **Condition** | **Starting material** | **Library Preparation approach** | **Sequencing** | **Analysis Sequence Clonetype** |
| --- | --- | --- | --- | --- | --- | --- | --- |
| SRR24889588 | *Hipposideros armiger* | Spleen | Healthy | RNA | 5’RACE | HTS | 512 |
| SRR24889587 | *Hipposideros armiger* | Spleen | Healthy | RNA | 5’RACE | HTS | 26512 |
| SRR24889586 | *Hipposideros armiger* | Spleen | Healthy | RNA | 5’RACE | HTS | 8600 |

**Supplemental Table S11. Basic information and unique TCRβ CDR3 sequences analyzed for all buffalo samples.**

| **Accession Number** | **Species** | **Tissue** | **Condition** | **Starting material** | **Library Preparation approach** | **Sequencing** | **Analysis Sequence Clonetype** |
| --- | --- | --- | --- | --- | --- | --- | --- |
| SRR24889447 | Buffalo | Spleen | Healthy | DNA | multiplex PCR | HTS | 29641 |
| SRR24889446 | Buffalo | Spleen | Healthy | DNA | multiplex PCR | HTS | 18623 |
| SRR24889445 | Buffalo | Spleen | Healthy | DNA | multiplex PCR | HTS | 15778 |
| SRR24889444 | Buffalo | Spleen | Healthy | DNA | multiplex PCR | HTS | 34392 |
| SRR24889443 | Buffalo | Spleen | Healthy | DNA | multiplex PCR | HTS | 13814 |
| SRR22523497 | Buffalo | Spleen | Healthy | DNA | multiplex PCR | HTS | 32118 |

**Supplemental Table S12. Basic information and unique TCRβ CDR3 sequences analyzed for all bovine samples.**

| **Accession Number** | **Species** | **Tissue** | **Condition** | **Starting material** | **Library Preparation approach** | **Sequencing** | **Analysis Sequence Clonetype** |
| --- | --- | --- | --- | --- | --- | --- | --- |
| SRR24889460 | Bovine | Spleen | Healthy | DNA | multiplex PCR | HTS | 33529 |
| SRR24889459 | Bovine | Spleen | Healthy | DNA | multiplex PCR | HTS | 18514 |
| SRR24889458 | Bovine | Spleen | Healthy | DNA | multiplex PCR | HTS | 42498 |
| SRR24889457 | Bovine | Spleen | Healthy | DNA | multiplex PCR | HTS | 27470 |
| SRR24889456 | Bovine | Spleen | Healthy | DNA | multiplex PCR | HTS | 21406 |
| SRR24889455 | Bovine | Spleen | Healthy | DNA | multiplex PCR | HTS | 17585 |
| SRR24889454 | Bovine | Spleen | Healthy | DNA | multiplex PCR | HTS | 18767 |
